# The BMI impact on thyroidectomy-related morbidity; a case-matched single institutional analysis

**DOI:** 10.1186/s12893-025-03018-0

**Published:** 2025-07-04

**Authors:** Sascha Vaghiri, Jasmin Mirheli, Dimitrios Prassas, Stephen Fung, Sami Alexander Safi, Georg Fluegen, Wolfram Trudo Knoefel, Levent Dizdar

**Affiliations:** 1 Department of Surgery (A), Medical Faculty, Heinrich-Heine-University, University Hospital Duesseldorf, Moorenstr. 5, 40225 Duesseldorf, Germany; 2 Medical Research School Duesseldorf, Heinrich-Heine-University Duesseldorf, Moorenstr. 5, 40225 Duesseldorf, Germany; 3 Department of Surgery, Katholisches Klinikum Essen, Philippusstift, Teaching Hospital of Duisburg-Essen University, Huelsmannstr, 17, 45355 Essen, Germany

**Keywords:** Obesity, Thyroid surgery, BMI, Postoperative complications

## Abstract

**Background:**

Obesity is associated with an increased risk of postoperative morbidity. We aimed to analyze the impact of BMI on surgical complications in patients undergoing thyroidectomy.

**Methods:**

This retrospective study was conducted in a single academic center. A total of 484 patients with open total thyroidectomy were considered eligible. These patients were divided in the non-obese (BMI < 30 kg/m^2^) and obese (BMI ≥ 30 kg/m^2^) groups. A 1:2 case matching based on demographic (age and gender) and clinical (benign/malignant disease) variables was performed to generate homogenous study groups. A comparative analysis was carried out to show the differences between the two groups in terms of the occurrence of surgery-related outcomes.

**Results:**

After case matching, 193 non-obese and 98 obese patients were included in the final analysis. There was no statistically significant difference in the rate of primary outcomes in the non-obese and obese groups: hypoparathyroidism (transient: 29% versus 21.4%, *p* = 0.166; permanent: 11.4% versus 15.3%, *p* = 0.344, respectively) and recurrent laryngeal nerve palsy (transient: 13.9% versus 11.2%, *p* = 0.498; permanent: 3.1% versus 2.0%, *p* = 0.594, respectively). A BMI ≥ 30 kg/m^2^ was associated with a significantly longer operative time (*p* = 0.018), while other secondary outcomes were not significantly affected by BMI.

**Conclusions:**

Despite prolonged operative times in obese patients, total thyroidectomy could be performed safely and without increased risk of surgery-related morbidity, regardless of BMI.

**Clinical trial number:**

Not applicable.

**Supplementary Information:**

The online version contains supplementary material available at 10.1186/s12893-025-03018-0.

## Background

Large epidemiological investigations indicate a high and increasing prevalence of obesity worldwide [1, 2]. It is estimated that in 2025 the global obesity prevalence will reach 18% in men and approximately 21% in women [3]. Obesity is significantly associated with a higher incidence of relevant comorbidities and conditions such as diabetes mellitus, all types of cancers except esophageal and prostate cancer, and cardiovascular and pulmonary disease [4, 5]. At the same time, treatment costs for obesity-related diseases are rising, which places an enormous burden on healthcare systems [6, 7]. Obesity is also linked to an increased risk of thyroid cancer in advanced stages (especially papillary thyroid carcinoma) and non-malignant thyroid disorders [8, 9, 10, 11]. As a result, surgeons are increasingly performing thyroid surgery on overweight patients, exposing themselves to the difficult surgical field of a short and circumferentially enlarged neck. In various surgical disciplines, a significantly higher rate of perioperative morbidity was found in obese compared to non-obese patients [12, 13, 14]. The most common surgical complications after thyroidectomy are hypocalcemia (3.5-14.5%, transient up to 28%) and recurrent laryngeal nerve (RLN) palsy (approximately 1-11%). Other complications such as wound hematoma and wound infection occur less frequently (< 1%) [15, 16, 17, 18, 19, 20, 21]. The extent to which body mass index (BMI) is a risk factor for postoperative complications after thyroid surgery is not fully understood, which is underlined by conflicting results in the existing literature. While some studies report no association between BMI and local complications, other authors demonstrated a significantly higher rate of thyroidectomy-specific morbidity in obese patients [22, 23, 24]. Therefore, the primary aim of this study was to assess the impact of BMI and in particular obesity on overall surgical morbidity and thyroidectomy-related complications such as RLN palsy and hypoparathyroidism in a single-center analysis.

## Methods

### Study design

Our prospectively maintained institutional database was used to identify and include all patients who underwent primary open total thyroidectomy for benign and malignant indications between January 2010 and July 2022 at the Surgical Department of the Heinrich-Heine-University, Duesseldorf, Germany. In the study protocol, the following exclusion criteria were considered: patient age < 18 years, missing or incomplete perioperative and follow-up data, hemi-thyroidectomies, and re-do or emergency procedures. The approval of the Institutional Review Board (IRB) at the Medical Faculty, Heinrich-Heine-University, Duesseldorf was granted (study-no.:2024–2789) prior study initiation. The study was conducted in strict adherence to the Strengthening the Reporting of Observational Studies in Epidemiology (STROBE) guidelines for observational studies [25] and the current ethical standards in the latest version of the Declaration of Helsinki. Informed consent was waived because no data regarding the cases were disclosed.

### Measurements

The following perioperative clinico-pathological information was extracted for each eligible patient: Preoperative: demographics including age, year, BMI, ASA score (American society of anesthesiologists), comorbidities, thyroid-related signs and symptoms, thyroid disease characteristics (e.g. malignant or benign), endocrinological laboratory and imaging findings.

Intraoperative: type and extend of surgery, intraoperative complications, visualization of the parathyroid glands, use of intraoperative neuromonitoring (IONM) and energy devices, and drainage placement.

Postoperative: laboratory values including calcium and parathyroid hormone (PTH), postoperative surgical morbidity, length of hospital stay, histological examination, and follow-up data.

### Endpoints and definitions

The primary study endpoints were postoperative RLN palsy and hypoparathyroidism, respectively. Transient RLN palsy was defined as vocal cord dysmotility based on the clinical examination (voice hoarseness and laryngoscopy) within a time period of 6 postoperative months, while permeant RLN palsy continues beyond 6 months after surgery. Postoperative clinical hypoparathyroidism was defined as total calcium levels less than the lower limit our center specific reference range (2.10–2.42 mmol/l) with or without hypocalcaemia symptoms, and the need of calcium and/or Vitamin D substitution in combination with undetectable or reduced PTH levels referenced by the laboratory standard (1.6–6.9 pmol/l) [26, 27]. Similar to RLN palsy, permanent hypoparathyroidism was diagnosed if clinical symptoms and abnormal biochemical findings persisted for more than 6 months after thyroidectomy [28]. Secondary outcomes of interest were operative time from skin incision to closure (minutes), postoperative hospital stays (days), bleeding complications (cervical hematoma and re-intervention), and wound infection. During hospital stay clinical and laboratory findings were evaluated on a daily basis until discharge. The follow-up examinations were carried out either in the form of outpatient appointments including laboratory checks or as a standardized phone interview specifically developed for this study (suppl. material). If necessary, the patients’ general practitioners were contacted to obtain follow-up information. At our department, vocal cord function was routinely evaluated pre- and postoperatively by laryngoscopy in all patients, not just those with clear clinical symptoms. However, we do not use transcutaneous laryngeal ultrasound, which is another easy-to-use and patient-friendly method for assessing RLN function [29]. In the case of RLN palsy, the patients were treated with speech therapy.

The study population was divided in two groups based on the suggested World Health Organization (WHO) classification of obesity: non-obese (BMI < 30 kg/m^2^) versus obese (BMI ≥ 30 kg/m^2^). Additionally, a third group of patients with obesity class ≥ II (BMI ≥ 35 kg/m^2^) was categorized [30]. In a further attempt to generate homogeneous and comparable groups, a 1:2 case matching of the obese and non-obese cohorts was performed according to demographic and clinical variables (gender, age ± 10 years, type of benign or malignant diagnosis).

### Statistical analysis

Continuous variables were reported as median (range) and assessed using the Mann-Whitney U test. Categorical data were summarized as frequencies (%) and compared using the chi-square test. In case of a comparative analysis involving more than two groups, the Kruskal-Wallis test was used. Post hoc statistical power regarding the primary and secondary outcomes was found to be as follows assuming a significance level of 0.05: transient hypoparathyroidism 28%, permanent hypoparathyroidism 15%, transient RLN palsy 10%, permanent RLN palsy 8%, operative time 59%, hospital stay 9%, cervical hematoma 5%, re-surgery 13%, and wound infection 11%. The difference in operative time between both obese and non-obese groups was visualized with a box plot figure. Statistical analysis was performed using G*Power [31] and the SPSS 25.0 software program (Statistical Package for Social Sciences; SPSS Inc., Chicago, IL, USA). A *p*-value < 0.05 represented the significance threshold.

## Results

### Patient selection and group characteristics

**Fig. 1:**
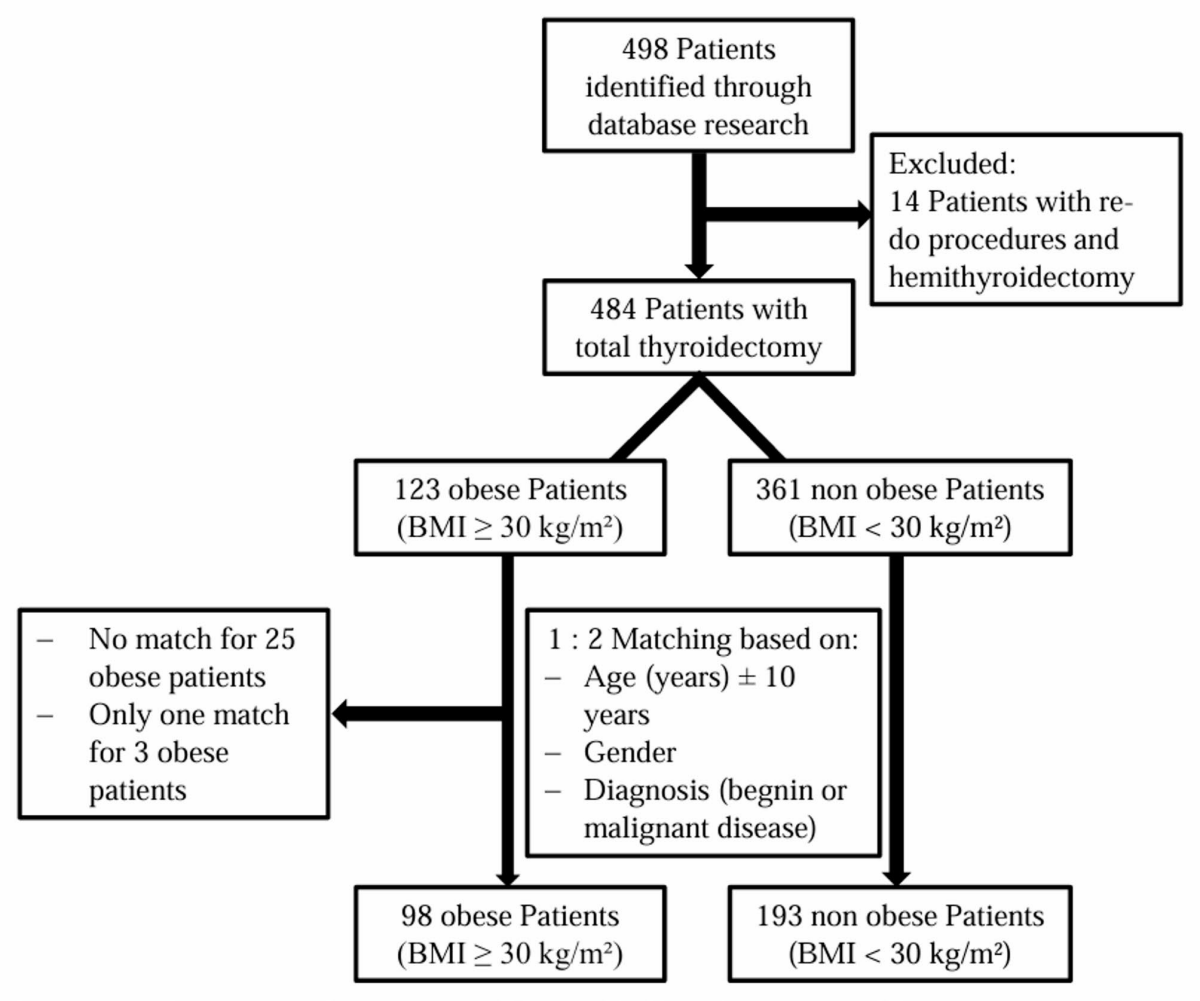
Flowchart diagram of patient selection and analysis

A total of 498 patients were identified by our database search. Based on the study criteria, 14 patients who underwent re-do procedures or hemithyroidectomies were consecutively excluded. The remaining 484 eligible patients with total open thyroidectomy were divided into obese (BMI ≥ 30 kg/m²; *n* = 123) and non-obese (BMI < 30 kg/m²; *n* = 361) cohorts based on their BMI (Fig. 1). A 1:2 case matching resulted in homogenous study groups of non-obese (*n* = 193) and obese (*n* = 98) patients with comparable demographic (age, gender) thyroid disease variables as illustrated in Table 1. Of note, the mean BMI ± SD in the non-obese group was 24.34 ± 3.26 kg/m^2^ compared to 34.4 ± 4.18 kg/m^2^ in obese patients. The maximum BMI-value recorded was 46.9 kg/m^2^ in our cohort. The majority of surgeries in both groups (non-obese 83.4% versus obese 82.7%) were performed due to benign thyroid conditions including Graves’ disease, goiter, and symptomatic Hashimoto thyroiditis. In the non-obese group, 52 patients (26.9%) underwent concomitant neck dissection compared to 32 patients (32.7%) in the obese group (Table 1.).

### Surgical outcomes

**Fig. 2:**
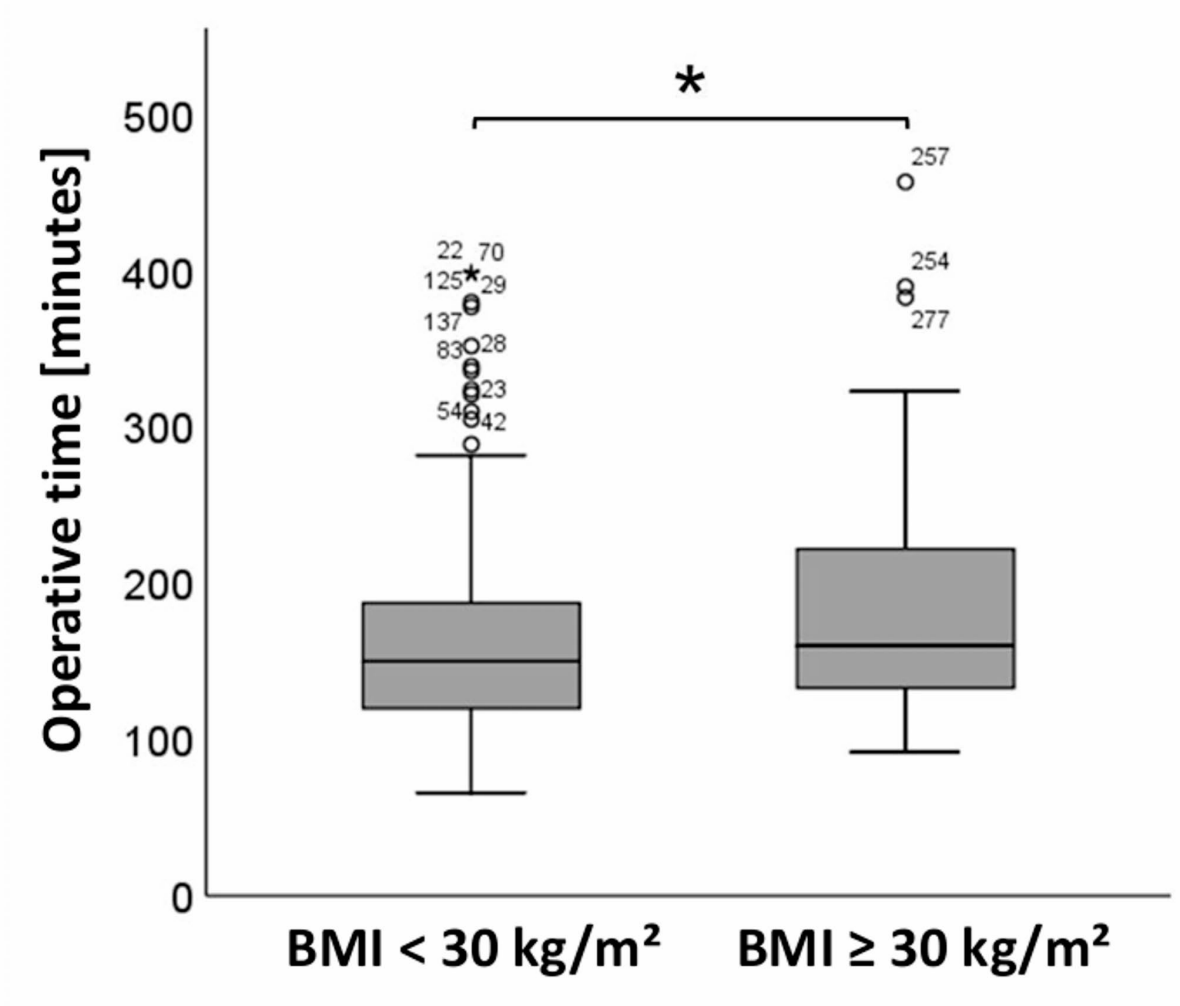
Boxplot visualization of operative times for non-obese and obese patients, * *p* = 0.032

Surgical outcomes are summarized in detail in Table 2. The median operative time was 10 min longer in the obese group [non-obese: 150 (range 66–399) minutes versus obese: 160 (range 92–457) minutes, respectively; *p* = 0.018] (Fig. 2), while the median hospital stay was not significantly different between both groups (*p* = 0.490). The rate of bleeding associated complications such as cervical hematoma (non-obese: 9.3% versus obese: 9.2%; *p* = 0.968) and need of re-surgery for hematoma evacuation (non-obese: 3.1% versus obese: 5.1%; *p* = 0.550) was equally distributed comparing both groups. In the whole study cohort, one wound infection in a non-obese patient was observed. Regarding our primary outcomes, the rate of postoperative hypoparathyroidism was not statistically different comparing both transient (non-obese 29% versus obese 21.4%; *p* = 0.166) and permanent (non-obese 11.4% versus obese 15.3%; *p* = 0.344) hypoparathyroidism. Furthermore, in both obese and non-obese patients, no statistically significantly different rates of transient (non-obese 13.9% versus obese 11.2%; *p* = 0.498), and permanent (non-obese 3.1% versus obese 2.0%; *p* = 0.594) RLN palsy were documented.

### Sub-analysis of patients with obesity

The obese group (BMI ≥ 30 kg/m^2^) was further subdivided into obesity class I (BMI 30–35 kg/m^2^) with 68 patients and obesity class II (BMI ≥ 35 kg/m^2^) encompassing 30 patients. Interestingly, we found that a significantly larger portion of patients in the obesity class II had thyroid cancer (33%) compared to only 10.3% in the obesity class I group and 16.7% in non-obese patients (*p* = 0.019). Other demographic and clinical variables did not differ significantly between obesity classes I and II patients (Table 3).

### Surgical outcomes for patients with obesity

Of note, in the obesity class I group operative time was significantly reduced compared to the obesity class II cohort [160 (92–390) minutes versus 165 (104–457) minutes; *p* = 0.032]. Other complications including hypoparathyroidism, RLN palsy, and hospital stay appeared to be unaffected by patient weight, especially with a focus on obesity classes I and II (Table 4).

## Discussion

The prevalence of obesity and thus the number of obese patients requiring surgical treatment is steadily increasing. Accordingly, endocrine surgeons are increasingly confronted with the challenges of severely overweight patients. In the context of thyroid surgery, these challenges include a short and circumferentially enlarged neck with a limited possibility of reclination, resulting in a reduced operating space. Whether these perceived obstacles by many surgeons lead to an actual worsening of surgical outcomes in obese patients is not consistently reported in the current literature [32, 33, 34, 35]. Thus, in the presented study we sought to elucidate the impact of obesity on surgically related outcomes after elective total thyroidectomy. Case matching led to homogeneity of the groups to be compared. Our analysis revealed that obesity (defined as BMI ≥ 30 kg/m^2)^ does not appear to influence the postoperative course with special regard to RLN palsy, hypoparathyroidism, bleeding, wound infection, and length of hospital stay. However, we noticed significantly longer surgery times in the cohort of obese patients (specifically obesity classes I and II) compared to the non-obese group.

Looking at the data from previously published studies that analyzed the influence of obesity on the outcome of patients undergoing thyroidectomy, most of these studies showed no effect of BMI on the incidence of surgical complications [33, 35, 36]. In line with these results, our analysis did not reveal any differences in surgical complications between the different BMI groups. The rates of RLN palsy and hypoparathyroidism in our analysis were comparable to results from large registry analyses [20, 21, 37], but were higher in comparison to recently published studies on the same subject from high-volume European and American centers [36, 38]. This observation could be due to several parameters in the study design and patient characteristics. In the study by Armstrong et al. [38] 22-35% of the patients underwent hemi-thyroidectomy while this rate was approximately 18% in the study of Rossi et al. [36]. In comparison, our study just included patients with total thyroidectomy in both BMI groups. Another important difference is the rate of thyroidectomy with concomitant neck dissection, which was about 26.9% in the non-obese group and 32.7% in the obese group, respectively. In contrast, Rossi et al. [36] only included 13% neck dissections in non-obese and 11% in obese patients while Armstrong et al. [38] did not consider patients with neck dissection in their study.

Another aspect that makes it difficult to compare the individual study results with each other is the considerable huge variation in definition and reported incidence of postsurgical hypoparathyroidism. A comprehensive review including 89 articles indicated that the incidence of hypoparathyroidism ranged from 0 to 20.2% based on 20 different definitions [39]. In our study, patients were stratified according to the recommended clinical hypoparathyroidism definition of the American Thyroid Association, which is based on biochemical, and clinical variables of hypothyroidism and hypocalcaemia [26]. Many studies analyzing BMI impact on surgical outcomes, referred only to hypocalcaemia and Calcium/vitamin D supplementation without taking into account parathyroid hormone levels [22, 24, 36, 37, 38]. Additionally, the follow-up period of hypoparathyroidism and/or hypocalcaemia was just limited to 30 days in some studies [37, 38]. Obesity is associated with vitamin D deficiency [40], which in turn makes patients with a high BMI more susceptible to postoperative hypocalcemia [41, 42]. Contrary to the assumption that obese people are more prone to postoperative hypocalcemia, one study found that higher BMI is protective against the risk of hypocalcemia after total thyroidectomy [37, 43]. The reason for this may be the reduced manipulative parathyroid trauma in the excessive covering fatty tissue [43]. On the other hand, the increased cervical adipose tissue could make the identification of both the parathyroid glands and the RLN considerably more difficult.

Obesity has been identified as a contributing factor to surgical site infection (SSI), which is a very rare complication after thyroidectomy at about 0.36% [44]. Consistent with this finding, the overall wound infection rate in our study was 0.34%. However, there was no significant difference in the rate of wound infection between non-obese and obese patients in our analysis. In contrast to this observation, Buerba et al. [32] and Chen et al. [34] found a significant correlation between a high BMI and an increased risk of wound complications after thyroidectomy in large patient cohorts. Due to the very low frequency of wound infections after thyroidectomies, it is likely that large numbers of cases are required to identify potential risk factors. Accordingly, it is possible that the number of cases we analyzed was too small to assess a correlation between BMI and the occurrence of wound infections after thyroidectomies. Consistent with the belief of many surgeons that obesity makes thyroid surgery more difficult, our analysis revealed that the operation time was significantly longer in patients with a BMI over 30 kg/m^2^ compared to non-obese patients. Buerba et al. [32] had similar results when analyzing data from the American College of Surgeons National Surgery Quality Improvement Program (ACS NSQIP). They found that the operating time was significantly prolonged with increasing BMI. In contrast to these observations a recent study by Rossi et al. [36] found no difference in operation times depending on the patients’ BMI. A possible explanation for this inconsistency could be the use of energy devices during surgery. While in our patient collective vascular closures were performed by ligation, Rossi et al. [36] used energy devices in about two-thirds of the operations. It must be assumed that the use of energy devices shortens the operating time in obese patients, as the often difficult tying of ligatures in the usually deep and narrowed surgical field is avoided. Beside surgical complications, thyroidectomy in obese patients was associated with an increased risk of adverse cardiopulmonary events, as reported in two large studies [34, 45].

Our study has some notable shortcomings with respect to its retrospective design that cannot be examined in a different fashion. Secondly, the relatively small sample sizes of both obese and non-obese cohorts combined with the low rate of some reported complications, limits adequate statistical power and conclusion as highlighted by the post hoc analysis. The present analysis only focused on local complications after total thyroidectomy, therefore, we cannot make any statements on the association between BMI and the general perioperative morbidity. Despite case matching and the formation of homogeneous and comparable study groups based on demographic and clinical data, a certain degree of bias could not be completely ruled out. Importantly the results presented here, were derived from an academic center over a period of more than 10 years, which may influence reproducibility for other institutions. In addition to BMI, other parameters such as the ratio of muscle mass to visceral fat or neck circumference could provide further valuable information on this topic. However, based on the available data, we were unable to include these values in our analysis.

## Conclusions

Thyroidectomy could be safely performed in obese patients with a similar rate of postoperative short-and long-term local complications compared to the non-obese population, but at the cost of prolonged operative time. However, to assess the impact of obesity and BMI on surgical and overall morbidity after thyroidectomy, larger multicenter trails with homogenous and standardized study protocols, and long-term follow-up data are needed.

**Table 1:** Demographic and clinical variables in non-obese and obese patients after case matching

	BMI < 30 kg/m^2^ (*n* = 193)	BMI ≥ 30 kg/m^2^ (*n* = 98)	*P*-value
Sex, n (%)			
Male	67 (34.7)	34 (34.7)	0.997
Female	126 (65.3)	64 (65.3)	
Age (years), median (range)	54 (21–80)	53 (21–79)	0.553
BMI kg/m^2^ (mean ± SD)	24.34 ± 3.26	34.43 ± 4.18	**< 0.0001**
Diagnosis, n (%)			
Benign disease	161 (83.4)	81 (82.7)	0.869
Malignant disease	32 (16.7)	17 (17.3)	
Type of surgery, n (%)			
Total thyroidectomy	141 (73.1)	66 (67.3)	0.310
Total thyroidectomy + neck dissection	52 (26.9)	32 (32.7)	

BMI: body max index, SD: standard deviation

**Table 2:** Surgical outcomes in non-obese and obese patients

	BMI < 30 kg/m^2^ (*n* = 193)	BMI ≥ 30 kg/m^2^ (*n* = 98)	*P*-value
Operative time (minutes), median (range)	150 (66–399)	160 (92–457)	**0.018**
Hospital stay (days), median (range)	3 (2–77)	3 (2–22)	0.490
Postoperative complications, n (%)			
Bleeding			
Cervical hematoma	18 (9.3)	9 (9.2)	0.968
Re-surgery	6 (3.1)	5 (5.1)	0.550
Wound infection	1 (0.5)	0	0.475
Hypoparathyroidism			
Transient	56 (29.0)	21 (21.4)	0.166
Permanent	22 (11.4)	15 (15.3)	0.344
RLN palsy			
Transient	27 (13.9)	11 (11.2)	0.498
Permanent	6 (3.1)	2 (2.0)	0.594

BMI: body mass index, RLN: recurrent laryngeal nerve

**Table 3:** Demographic, clinical, and surgical variables for obesity classes I and II patients

	BMI 30–35 kg/m^2^ (*n* = 68)	BMI ≥ 35 kg/m^2^ (*n* = 30)	*P*-value
Sex, n (%)			
Male	25 (36.8)	9 (30.0)	0.810
Female	43 (63.2)	21 (70.0)	
Age (years), median (range)	52 (21–76)	53 (27–79)	0.813
Diagnosis, n (%)			
Benign disease	61 (89.7)	20 (66.7)	**0.019**
Malignant disease	7 (10.3)	10 (33.3)	
Type of surgery, n (%)			
Total thyroidectomy	48 (70.6)	18 (60.0)	0.338
Total thyroidectomy + neck dissection	20 (29.4)	12 (40.0)	

BMI: body mass index

**Table 4:** Surgical outcomes for obesity classes I and II patients

	BMI 30–35 kg/m^2^ (*n* = 68)	BMI ≥ 35 kg/m^2^ (*n* = 30)	*P*-value
Operative time (minutes), median (range)	160 (92–390)	165 (104–457)	**0.032**
Hospital stay (days), median (range)	3 (2–22)	3 (2–16)	0.468
Postoperative complications, n (%)			
Bleeding			
Cervical hematoma	5 (7.4)	4 (13.3)	0.642
Re-surgery	3 (4.4)	2 (6.7)	0.517
Wound infection	0	0	0.775
Hypoparathyroidism			
Transient	14 (20.6)	7 (23.3)	0.367
Permanent	11 (16.1)	4 (13.3)	0.593
RLN palsy			
Transient	8 (11.8)	3 (10.0)	0.773
Permanent	2 (2.9)	0	0.620

BMI: body mass index, RLN: recurrent laryngeal nerve

## Electronic supplementary material

Below is the link to the electronic supplementary material.

Supplementary Material 1

Supplementary Material 2

## Data Availability

The datasets used and/or analyzed during the current study are available from the corresponding author on reasonable request.
