# Supplementary material for: The BMI impact on thyroidectomy-related morbidity; a case-matched single institutional analysis
Source: BMC Surg. 2025 Jul 4;25:286. doi: 10.1186/s12893-025-03018-0 (PMC12231610; doi:10.1186/s12893-025-03018-0)
Supplement: Supplementary file 1 — Supplementary Material 1 [file 12893_2025_3018_MOESM1_ESM.docx]

STROBE Statement—checklist of items that should be included in reports of observational studies

|  | Item No. | Recommendation | Page  No. | Relevant text from manuscript |
| --- | --- | --- | --- | --- |
| **Title and abstract** | 1 | (*a*) Indicate the study’s design with a commonly used term in the title or the abstract | Tile page 1 | The BMI impact on thyroidectomy related morbidity; a case-matched single institutional analysis. |
|  |  | (*b*) Provide in the abstract an informative and balanced summary of what was done and what was found | Abstract page 2 |  |
| Introduction | | | |  |
| Background/rationale | 2 | Explain the scientific background and rationale for the investigation being reported | Introduction page 3 | The extent to which body mass index (BMI) is a risk factor for postoperative complications after thyroid surgery is not fully understood, which is underlined by conflicting results in the existing literature (22–24). Therefore, the primary aim of this study was to assess the impact of BMI and in particular obesity on postoperative morbidity after thyroidectomy in a single-center analysis. |
| Objectives | 3 | State specific objectives, including any prespecified hypotheses | Introduction page 3 |  |
| Methods | | | |  |
| Study design | 4 | Present key elements of study design early in the paper | Methods pages 3-5 | Retrospective, single center study , case matched study population |
| Setting | 5 | Describe the setting, locations, and relevant dates, including periods of recruitment, exposure, follow-up, and data collection | Methods pages 3-5 |  |
| Participants | 6 | (*a*) *Cohort study*—Give the eligibility criteria, and the sources and methods of selection of participants. Describe methods of follow-up  *Case-control study*—Give the eligibility criteria, and the sources and methods of case ascertainment and control selection. Give the rationale for the choice of cases and controls  *Cross-sectional study*—Give the eligibility criteria, and the sources and methods of selection of participants | Methods pages 3-5 | Our prospectively maintained institutional database was used to identify and include all patients who underwent primary open thyroidectomy for benign and malignant indications between January 2010 and July 2022 at the Surgical Department of the Heinrich-Heine-University, Duesseldorf, Germany. In the study protocol, the following exclusion criteria were considered: patient age < 18 years, missing or incomplete perioperative and follow-up data, hemi-thyroidectomies, and re-do or emergency procedures. |
|  |  | (*b*) *Cohort study*—For matched studies, give matching criteria and number of exposed and unexposed  *Case-control study*—For matched studies, give matching criteria and the number of controls per case | Methods pages 3-5 | In a further attempt to generate homogeneous and comparable groups, case matching of the obese and non-obese cohorts was performed according to demographic and clinical variables (sex, age, type of diagnosis and surgery). |
| Variables | 7 | Clearly define all outcomes, exposures, predictors, potential confounders, and effect modifiers. Give diagnostic criteria, if applicable | Methods pages 3-5 | The primary study endpoints were postoperative RLN palsy and hypoparathyroidism, respectively. Transient RLN palsy was defined as vocal cord dysmotility based on the clinical examination (voice hoarseness and laryngoscopy) within a time period of 6 postoperative months, while permeant RLN palsy continues beyond 6 months after surgery. |
| Data sources/ measurement | 8* | For each variable of interest, give sources of data and details of methods of assessment (measurement). Describe comparability of assessment methods if there is more than one group | Methods pages 3-5 |  |
| Bias | 9 | Describe any efforts to address potential sources of bias | Methods pages 3-5 | Case matching was perfomed to reduce potential sources of bias |
| Study size | 10 | Explain how the study size was arrived at | Methods pages 3-5 | Retrospective study cohort , case matching |

Continued on next page

| Quantitative variables | 11 | Explain how quantitative variables were handled in the analyses. If applicable, describe which groupings were chosen and why | Methods pages 3-5 | The study population was divided in two groups based on the suggested World Health Organization (WHO) classification of obesity: non-obese (BMI < 30 kg/m2) versus obese (BMI ≥ 30 kg/m2). Additionally, a third group of patients with obesity class ≥ II (BMI ≥ 35 kg/m2) was categorized (29). |
| --- | --- | --- | --- | --- |
| Statistical methods | 12 | (*a*) Describe all statistical methods, including those used to control for confounding | Methods pages 4-5 | Continuous variables were reported as median (range) and assessed using the Mann-Whitney U test. Categorical data were summarized as frequencies (%) and compared using the chi-square test. In case of a comparative analysis involving more than two groups, the Kruskal-Wallis test was used. The difference in operative time between both obese and non-obese groups was visualized with a box plot figure. Statistical analysis was performed with the SPSS 25.0 software program (Statistical Package for Social Sciences; SPSS Inc., Chicago, IL, USA). A p-value < 0.05 represented the significance threshold. |
|  |  | (*b*) Describe any methods used to examine subgroups and interactions | Methods pages 3-5 |  |
|  |  | (*c*) Explain how missing data were addressed |  |  |
|  |  | (*d*) *Cohort study*—If applicable, explain how loss to follow-up was addressed  *Case-control study*—If applicable, explain how matching of cases and controls was addressed  *Cross-sectional study*—If applicable, describe analytical methods taking account of sampling strategy | Methods pages 3-5 | During hospital stay clinical and laboratory findings were evaluated on a daily basis until discharge. The follow-up examinations were carried out either in the form of outpatient appointments including laboratory checks or as telephone interviews. If necessary, the patients’ general practitioners were contacted to obtain follow-up information. |
|  |  | (*e*) Describe any sensitivity analyses | NA |  |
| Results | | | | |
| Participants | 13* | (a) Report numbers of individuals at each stage of study—eg numbers potentially eligible, examined for eligibility, confirmed eligible, included in the study, completing follow-up, and analysed | Results  page 5 | A total of 498 patients with total thyroidectomy were eligible for the analysis, including 123 obese patients (24.7%) with a BMI ≥ 30 kg/m2. Fourteen patients who underwent re-do procedures or hemithyroidectomies were consecutively excluded. Case matching resulted in homogenous study groups of non-obese (n = 193) and obese (n = 98) patients with comparable demographic (age, sex) and clinical variables (benign or malignant disease and thyroidectomy +/- neck dissection) as illustrated in table 1. |
|  |  | (b) Give reasons for non-participation at each stage | Results  page 5 | Fourteen patients who underwent re-do procedures or hemithyroidectomies were consecutively excluded. |
|  |  | (c) Consider use of a flow diagram | NA |  |
| Descriptive data | 14* | (a) Give characteristics of study participants (eg demographic, clinical, social) and information on exposures and potential confounders | Results  page 5 , tables 1 and 3 |  |
|  |  | (b) Indicate number of participants with missing data for each variable of interest | NA |  |
|  |  | (c) *Cohort study*—Summarise follow-up time (eg, average and total amount) | Results  page 5 |  |
| Outcome data | 15* | *Cohort study*—Report numbers of outcome events or summary measures over time | Results  page 5 , tables 2 and 4 |  |
|  |  | *Case-control study—*Report numbers in each exposure category, or summary measures of exposure | *NA* |  |
|  |  | *Cross-sectional study—*Report numbers of outcome events or summary measures | *NA* |  |
| Main results | 16 | (*a*) Give unadjusted estimates and, if applicable, confounder-adjusted estimates and their precision (eg, 95% confidence interval). Make clear which confounders were adjusted for and why they were included | Results  page 5 , tables 2 and 4 |  |
|  |  | (*b*) Report category boundaries when continuous variables were categorized | NA |  |
|  |  | (*c*) If relevant, consider translating estimates of relative risk into absolute risk for a meaningful time period | NA |  |

Continued on next page

| Other analyses | 17 | Report other analyses done—eg analyses of subgroups and interactions, and sensitivity analyses | Results  page 5 , tables 3,4 | Subgroup of Obesity class I and II |
| --- | --- | --- | --- | --- |
| Discussion | | | | |
| Key results | 18 | Summarise key results with reference to study objectives | Discussion page 6 | Our analysis revealed that obesity (defined as BMI ≥ 30 kg/m2) does not appear to influence the postoperative course with special regard to RLN palsy, hypoparathyroidism, bleeding, wound infection, and length of hospital stay. However, we noticed significantly longer surgery times in the cohort of obese patients (specifically obesity classes I and II) compared to the non-obese group. |
| Limitations | 19 | Discuss limitations of the study, taking into account sources of potential bias or imprecision. Discuss both direction and magnitude of any potential bias | Discussion pages 7,8 | Our study has some notable shortcomings with respect to its retrospective design that cannot be examined in a different fashion. Secondly, the relatively small sample sizes of both obese and non-obese cohorts combined with the low rate of some reported complications, potentially limits adequate statistical power analysis and conclusion. The present analysis only focused on local complications after thyroidectomy, therefore, we cannot make any statements on the association between BMI and the general perioperative morbidity. Despite case matching and the formation of homogeneous and comparable study groups based on demographic and clinical data, a certain degree of bias could not be completely ruled out. Importantly the results presented here, were derived from an academic teaching center over a period of more than 10 years, which may influence reproducibility for other institutions. |
| Interpretation | 20 | Give a cautious overall interpretation of results considering objectives, limitations, multiplicity of analyses, results from similar studies, and other relevant evidence | Discussion pages 6,7 |  |
| Generalisability | 21 | Discuss the generalisability (external validity) of the study results | Discussion pages 6,7 |  |
| Other information | |  | | |
| Funding | 22 | Give the source of funding and the role of the funders for the present study and, if applicable, for the original study on which the present article is based | Statements and Declarations page 9 | Open Access funding enabled and organized by Projekt DEAL. |

*Give information separately for cases and controls in case-control studies and, if applicable, for exposed and unexposed groups in cohort and cross-sectional studies.

**Note:** An Explanation and Elaboration article discusses each checklist item and gives methodological background and published examples of transparent reporting. The STROBE checklist is best used in conjunction with this article (freely available on the Web sites of PLoS Medicine at http://www.plosmedicine.org/, Annals of Internal Medicine at http://www.annals.org/, and Epidemiology at http://www.epidem.com/). Information on the STROBE Initiative is available at www.strobe-statement.org.
