# Supplementary material for: The BMI impact on thyroidectomy-related morbidity; a case-matched single institutional analysis
Source: BMC Surg. 2025 Jul 4;25:286. doi: 10.1186/s12893-025-03018-0 (PMC12231610; doi:10.1186/s12893-025-03018-0)
Supplement: Supplementary file 2 — Supplementary Material 2 [file 12893_2025_3018_MOESM2_ESM.docx]

**Standardized phone interview for this study:**

Have you experienced any complications such as a hematoma, wound infection, accumulation of fluid or other complaints after thyroid surgery?

Did you experience any dysfunction of the vocal cords after the operation?

If yes, how long did the symptoms persist?

Are you still seeing an endocrinology specialist or your general practitioner?

Did you develop hypoparathyroidism or hypocalcemia after the operation? Did you take calcium or vitamin D regularly after the operation? If yes, how long did the symptoms last and how long was the treatment required?
